# Allele-Specific Transcriptome and Methylome Analysis Reveals Stable Inheritance and *Cis*-Regulation of DNA Methylation in *Nasonia*

**DOI:** 10.1371/journal.pbio.1002500

**Published:** 2016-07-05

**Authors:** Xu Wang, John H. Werren, Andrew G. Clark

**Affiliations:** 1 Department of Molecular Biology and Genetics, Cornell University, Ithaca, New York, United States of America; 2 Cornell Center for Comparative and Population Genomics, Cornell University, Ithaca, New York, United States of America; 3 Department of Biology, University of Rochester, Rochester, New York, United States of America; Institute of Science and Technology Austria (IST Austria), AUSTRIA

## Abstract

Gene expression divergence between closely related species could be attributed to both *cis-* and *trans-* DNA sequence changes during evolution, but it is unclear how the evolutionary dynamics of epigenetic marks are regulated. In eutherian mammals, biparental DNA methylation marks are erased and reset during gametogenesis, resulting in paternal or maternal imprints, which lead to genomic imprinting. Whether DNA methylation reprogramming exists in insects is not known. Wasps of the genus *Nasonia* are non-social parasitoids that are emerging as a model for studies of epigenetic processes in insects. In this study, we quantified allele-specific expression and methylation genome-wide in *Nasonia vitripennis* and *Nasonia giraulti* and their reciprocal F1 hybrids. No parent-of-origin effect in allelic expression was found for >8,000 covered genes, suggesting a lack of genomic imprinting in adult *Nasonia*. As we expected, both significant *cis-* and *trans-* effects are responsible for the expression divergence between *N*. *vitripennis* and *N*. *giraulti*. Surprisingly, all 178 differentially methylated genes are also differentially methylated between the two alleles in F1 hybrid offspring, recapitulating the parental methylation status with nearly 100% fidelity, indicating the presence of strong *cis-*elements driving the target of gene body methylation. In addition, we discovered that total and allele-specific expression are positively correlated with allele-specific methylation in a subset of the differentially methylated genes. The 100% *cis*-regulation in F1 hybrids suggests the methylation machinery is conserved and DNA methylation is targeted by *cis* features in *Nasonia*. The lack of genomic imprinting and parent-of-origin differentially methylated regions in *Nasonia*, together with the stable inheritance of methylation status between generations, suggests either a *cis*-regulatory motif for methylation at the DNA level or highly stable inheritance of an epigenetic signal in *Nasonia*.

## Abstract 2

RNA-sequencing and whole-genome bisulfite sequencing in the hybrid offspring of two *Nasonia* parasitoid wasp species revealed strong *cis*-regulation of methylation and allele-specific expression. No gene was found to display genomic imprinting.

## Author Summary

The relationship between methylation of genomic DNA and expression of the genes that it encodes—and how this relationship changes during evolution—has been widely studied in mammals, but remains less well understood for insects. Here we analyze the expressed mRNA transcripts and genomic DNA methylation of the hybrid offspring of a pair of *Nasonia* parasitoid wasp species, producing a wealth of information about the regulation of gene expression. We find that variation in DNA sequence impacts expression on the same strand (called “*cis-*regulation”), and that cytosine methylation state is also associated in *cis* with the regulatory consequences of this base alteration. We show that these wasp species lack differential expression dependent on parent-of-origin (called “genomic imprinting”), and that in the hybrids the alleles retain the methylation status of the parental species in a strong *cis*-regulated fashion. Transcript abundances were also largely driven in a *cis*-regulated manner, consistent with a correlation between methylation status and expression levels. Despite the many differences between *Nasonia* and mammals in the impact of genomic DNA methylation, in both groups the use of methylated cytosine has been co-opted in ways that help tune gene expression.

## Introduction

Expression divergence between orthologous genes in closely related species could be attributed to species-specific DNA sequence changes in *cis*-regulatory elements and/or *trans*-factors. *Cis*-regulatory changes in promoter, enhancer, or 3′-UTR regions can alter expression by changes in transcription initiation or transcript stability [1], and sequence changes in *trans*-regulatory factors may also result in altered transcription rates. Interspecific F1 hybrids provide an excellent system to study the *cis*- versus *trans*-effects, and previous studies have shown that both significant *cis*- and *trans*-effects are present in yeast [2], *Arabidopsis* [3], *Drosophila* [4], mouse [5], and human [6].

For animal species with genome-wide DNA methylation, it remains an open question to determine if differential methylation between closely related species drives differences in gene expression, and whether it is allele-specific (*cis*-acting). To address this, we need a system with relatively stable DNA methylation and a method to quantify genome-wide allele-specific methylation in F1 hybrids with high accuracy. The bulk of CpG methylation in mammals occurs in intergenic regions [7], while *Drosophila* lacks symmetrical CpG methylation [8,9]. Eusocial insects such as honey bees and ants have gene body DNA methylation [10–14], but caste-specific methylation patterns are often found, necessitating a careful control of caste in experimental designs [15,16]. In addition, there are substantial DNA polymorphisms in social insects that can complicate DNA methylation calling.

The parasitoid wasp genus *Nasonia* is emerging as an excellent model for DNA methylation studies in insects [17]. Two closely related species, *Nasonia vitripennis* (Nv) and *Nasonia giraulti* (Ng) diverged about 1 million years ago and the synonymous coding divergence is ~3% [18]. About one-third of the genes in the *Nasonia* genome are methylated, and the bulk of methylated CpGs are found in the first kilobase of the coding region of these genes [19]. Between 150 and 200 genes are significantly differentially methylated between the two species, and almost no difference in methylation was found between the two sexes within species [20]. *Nasonia* is inbred in nature, and the depressed level of polymorphism within species provides great advantages for accurately assigning parental origin of the bisulfite sequencing reads from F1 hybrids, allowing more precise inference of allele-specific methylation. In this study, we performed RNA-seq and whole-genome bisulfite sequencing (WGBS-seq) in reciprocal F1 adult samples of Nv and Ng quantifying both total and allele-specific expression and DNA methylation genome-wide. With the RNA-seq and WGBS-seq data previously generated for the two parental species [19,20], we investigated the *cis*- versus *trans*- regulatory changes during evolution for both gene expression and DNA methylation, and we determined whether sequence changes affect both expression and epigenetic divergence.

A form of imprinting that results in paternal chromosome loss was first reported in insects [21–25]. However, subsequent studies in mammals revealed a different and widespread form of genomic imprinting, alterations of allelic expression depending on whether the allele was maternal or paternal in origin [26]. In animals, this form of genomic imprinting is only known to occur in therian mammals, including marsupials [27,28].

Studies in mammals revealed that the imprinting status of the parental alleles is marked at the imprinting control regions (ICRs), which often involve parent-of-origin dependent differentially methylated regions (pDMRs) [29]. Parental alleles can be active or repressed depending on the location of the pDMRs [7]. In principle, genomic imprinting could originate in any species with DNA methylation machinery able to transmit different methylation states through the male and female germline. The high rate of turnover of imprinting status between human, mouse, and equine species [30] suggests that there is a fairly high lability of imprinting status in placental mammals. The presence of imprinting quantitative trait loci (iQTLs) suggests genomic imprinting might exist in chickens [31,32]. However, two recent studies in chickens did not find any imprinted genes in either adults [33] or embryos [34]. To our knowledge, imprinted genes have not yet been found in other vertebrate species beyond therian mammals, despite the fact that many of the evolutionary arguments for the origin of genomic imprinting through genomic conflict ought to hold outside of mammals [35].

In insects, *Drosophila* lacks DNMT-dependent CpG methylation due to loss of DNA methyltransferases (*Dnmt*) 1 and 3 [8], with only low levels of asymmetrically methylated CpGs induced by other mechanisms [36,37], and no imprinted genes have been found in *Drosophila* whose genome was not perturbed by unnatural rearrangements [38,39]. Other insect species, including hymenopterans, do have both *Dnmt1* and *Dnmt3* as well as genome-wide CpG methylation [40], which provides epigenetic regulatory potential for the origin and maintenance of genomic imprinting. In eusocial insects with haplodiploid sex determination (e.g., social wasps, bees, and ants), theory predicts that sexual and social caste conflict could lead to parent-of-origin effects of allelic expression in F1s [41–44]. Several recent studies have discovered evidence suggestive of the presence of genomic imprinting in bumble bees [45,46] and honey bees [47]; however, a recent study has raised issues concerning the reliability of methylation estimates [15]. *Nasonia* have several advantages for quantification of methylation and expression differences within species and hybrids. Foremost, natural inbred lines in this insect considerably reduce the problems of mapping methylation due to the paucity of SNP variation within lines. Secondly, the availability of reliably mapped SNP differences between species permits genome-wide identification of both allele-specific gene expression and allele-specific methylation patterns in F1 hybrids. We utilize these advantages to determine whether there is any whole body parent-of-origin differential expression (genomic imprinting) in this non-eusocial species, and whether allele-specific differences in methylation are inherited in F1 progeny. The results and comparison with eusocial insects can shed light on the evolution and mechanisms of parent-of-origin expression and methylation in insects.

To interpret the results reported below, an understanding of haplodiploid genetic systems is crucial. In *Nasonia* (as other haplodiploids), females are derived from fertilized eggs and are diploid, whereas males are derived from unfertilized eggs and are haploid. Therefore, studies of allele-specific expression and allele-specific methylation are conducted exclusively in diploid females.

## Results

### Lack of Parent-of-Origin Differential Allelic Expression (pDAE) in Adult Reciprocal F1 Hybrids

**Fig 1:**
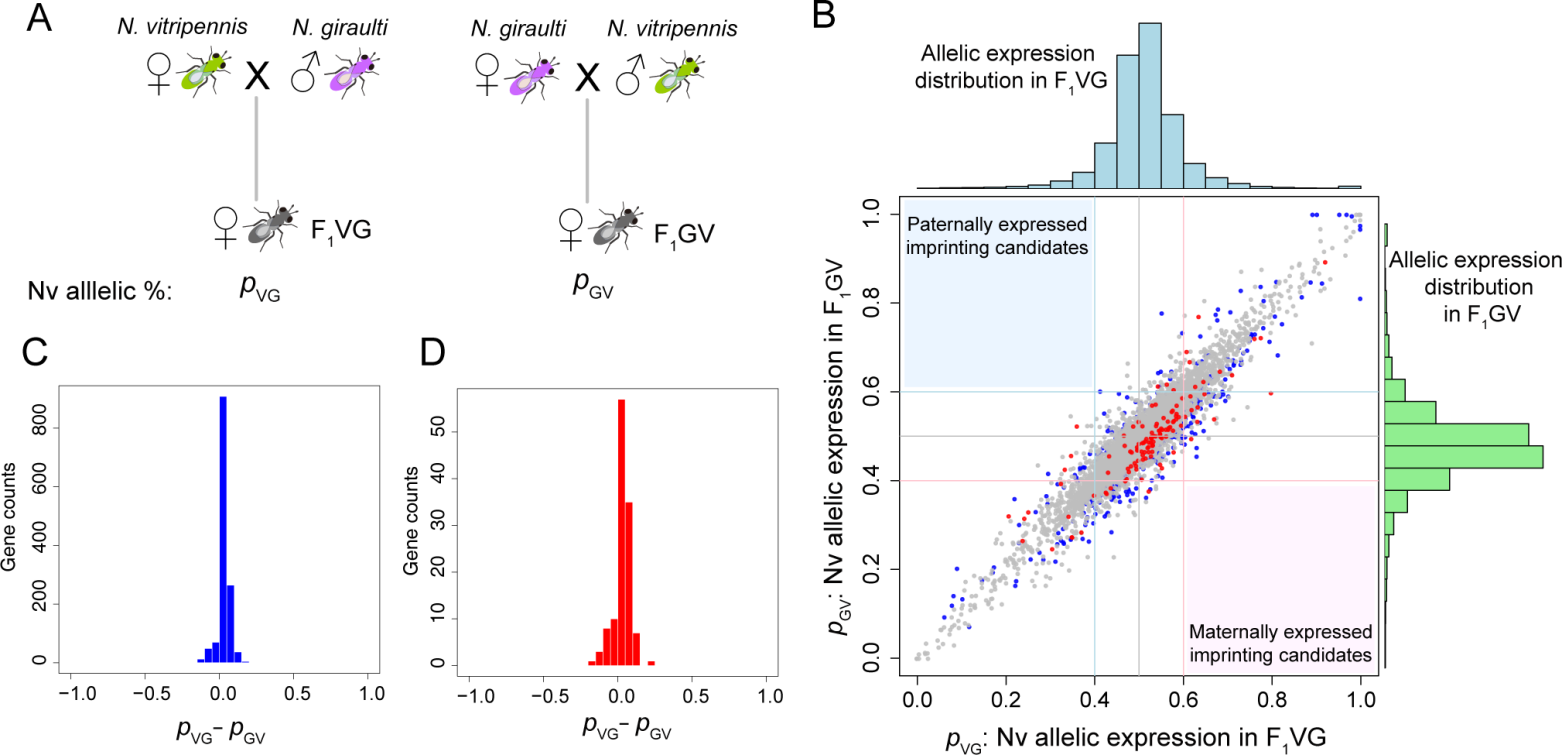
Lack of significant parent-of-origin allelic expression in reciprocal F1 crosses of *Nasonia vitripennis* and *Nasonia giraulti*. (***A***) Scheme of reciprocal F1 crosses between *N*. *vitripennis* (Nv) and *N*. *giraulti* (Ng). The Nv allelic expression percentage is quantified by *p*_VG_ in Nv mother x Ng father cross (F_1_VG), and *p*_GV_ in Ng mother x Nv father (F_1_GV). (***B***) Scatterplot of joint distribution of allelic expression percentage *p*_VG_ (*x*-axis) and *p*_GV_ (*y*-axis), for 8,623 covered genes with two or more high quality informative SNPs between Nv and Ng. Genes with significant allelic expression difference were plotted in blue (significant in all sample combined, FDR < 0.01, Fisher’s exact test) and red (significant and homogeneous in all three biological replicates; *p*-value < 0.01, replicated G-test of independence). Paternally expressed imprinting candidate genes should appear in the upper left corner (light-blue–shaded region), and maternal ones in the lower right corner (pink-shaded region). Histograms of *p*_VG_ (blue) and *p*_GV_ (green) were shown in the top and the right panel, respectively. (**C**) Histogram of allelic expression differences (*p*_VG_—*p*_GV_) for significant genes in all samples combined (blue dots in **B**). (**D**) Histogram of allelic expression differences (*p*_VG_—*p*_GV_) for significant genes in all biological replicates (red dots in **B**). Data presented in this figure can be found at http://dx.doi.org/10.5061/dryad.qf2t8.

To identify parent-of-origin effects in gene expression, we quantified gene expression levels transcriptome-wide in Nv and Ng [20] as well as allele-specific expression (ASE) in their reciprocal F1 hybrid daughters (F_1_VG: Nv mother x Ng father; F_1_GV: Ng mother x Nv father; Fig 1A). The experiments utilized RNA-seq in whole adult samples of three independent biological replicates per cross (Materials and Methods). Among 12,268 genes with detectable expression levels (fragments per kilobase of transcript per million mapped reads [FPKM] > 0.5) in both F1s, 8,623 have two or more high quality SNPs (combined coverage > 60X in all replicates) to accurately score allele-specific expression. The proportion Nv allelic expression was quantified at these informative SNP positions from RNA-seq data in F_1_VG (*p*_VG_) and F_1_GV (*p*_GV_). *p*_VG_ and *p*_GV_ have remarkably high correlation (Spearman’s rho = 0.91; Fig 1B). For all replicates combined, the average absolute reciprocal cross allelic expression difference is just 4% (mean |*p*_VG_—*p*_GV_| = 0.04; Fig 1C). These small allelic expression differences have large variance among replicates, and the variance was found to exceed binomial variance. Although a standard G-test indicated that 122 genes had *p* < 0.01 for the test of homogeneity of *p*_*VG*_ and *p*_*GV*_, (Fig 1B and 1D), a beta-binomial test that accommodates the over-dispersion fails to find any of these to be significant. Furthermore, even if we use a less stringent definition of paternally or maternally expressed imprinting candidates as genes with greater than 60% of paternal or maternal allelic expression in both F_1_VG and F_1_GV crosses [48], not a single candidate imprinted gene can be detected among more than 8,600 covered genes (Fig 1B). Therefore we conclude that there is no evidence of genomic imprinting in *Nasonia* whole adult female samples. This does not preclude imprinting in specific tissues or different life stages, however.

### Presence of Both *Cis-* and *Trans-* Effects on Gene Expression Differences between *N*. *vitripennis* and *N*. *giraulti*

**Fig 2:**
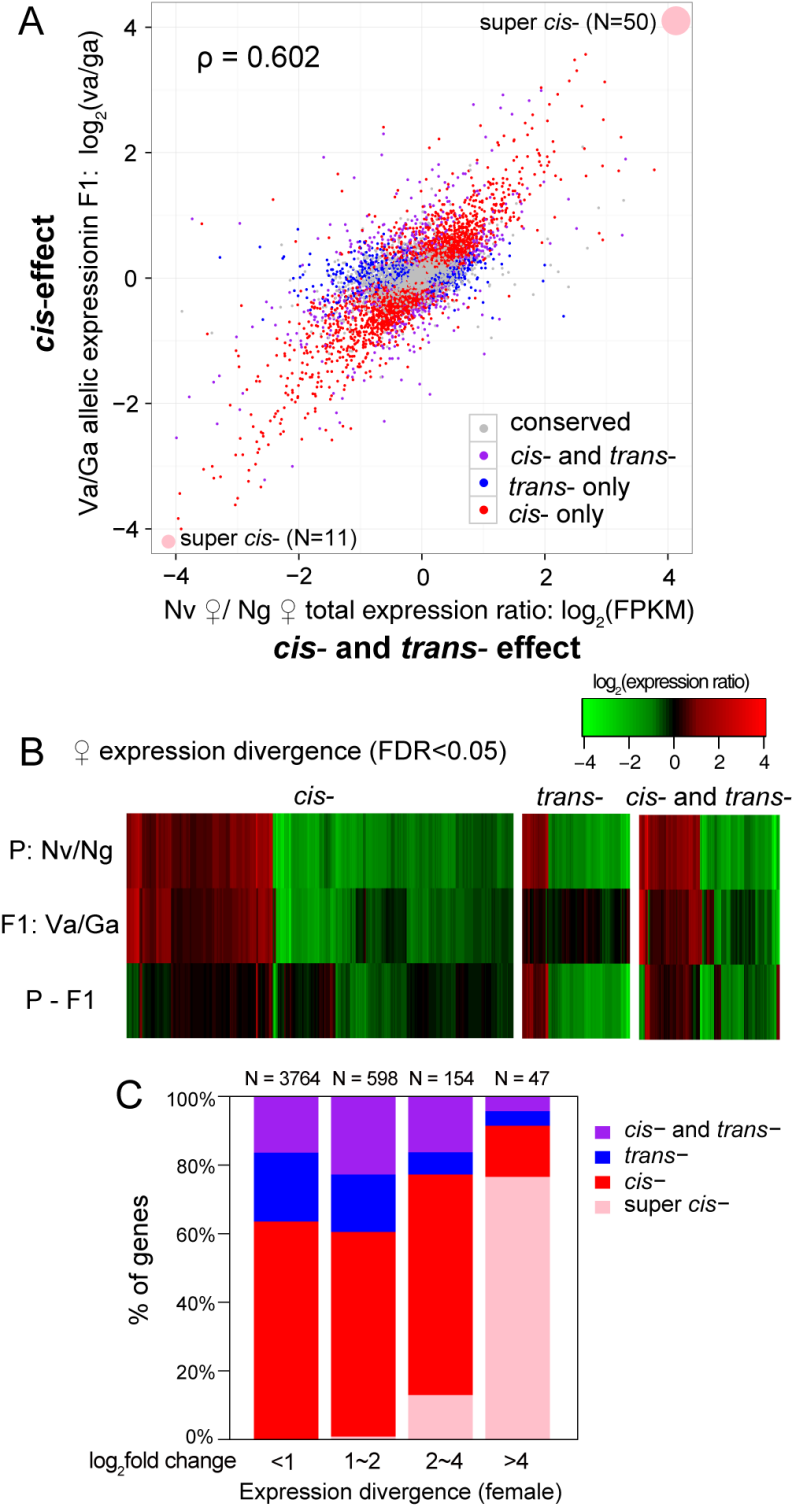
Relationship between *cis*- and *trans*- effect and expression divergence between *N*. *vitripennis* and *N*. *giraulti*. **(A)** Scatter plot of *N*. *vitripennis* and *N*. *giraulti* female total expression ratios (Nv/Ng) on the *x*-axis against average relative allelic expression from *vitripennis* allele (Va) and *giraulti* allele (Ga) in F1s on the *y*-axis. Conserved genes with no significant *cis*- or *trans*- effect are labeled in gray. *cis*- only, *trans*- only, and *cis*- and *trans*- genes are labeled in red, blue, and purple, respectively. Spearman's rank correlation coefficient ρ is labeled in the top left corner. Sixty-one genes with 100% monoallelic expression in at least one F1 sample are defined as super *cis-* genes and are represented by pink dots in the top right and bottom left corner. **(B)** Heatmap of genes with significant *cis*-, *trans-*, and *cis*- and *trans*- effects on female gene expression divergence. Only genes with significant female expression difference between *N*. *vitripennis* and *N*. *giraulti* are included (FDR < 0.05). **(C)** Stacked barplot showing the distribution of *cis*-, *trans*-, *cis*- and *trans*- and super *cis*- class genes for different categories of expression fold differences between *N*. *vitripennis* and *N*. *giraulti* for female expression divergence. Data presented in this figure can be found at http://dx.doi.org/10.5061/dryad.qf2t8.

The contribution of *cis*-regulatory factors to expression divergence between *N*. *girault*i and *N*. *vitripennis* can be quantified as log_2_(V/G allelic expression in F1s) and the *trans-*effect can be expressed as the expression divergence (log_2_ Nv/Ng total expression in parental species) minus the *cis-*effect [3] (S1 Fig). Since the two reciprocal F1s have an extremely high correlation in total gene expression (Spearman's rank correlation coefficient ρ = 0.987; S2 Fig) as well as allelic expression level (Fig 1B), we treated the replicates from both F1 crosses the same when we determined the following four classes: genes with no significant *cis*- or *trans*- effect (conserved), genes with significant *cis*- but not *trans*- effect (*cis*-), genes with significant *trans*- but not *cis*- effect (*trans*-) and genes with both significant *cis*- and *trans*- effect (*cis*- and *trans*-) (Fig 2A). Because the diploid F1s are all females, we used expression levels of the Nv and Ng females to quantify the expression divergence of the two parental species (Fig 2). 61 genes display 100% monoallelic expression in at least one F1 sample (S1 Table). We checked the total expression ratios in the two parental species and all of these genes displayed strong *cis*-regulation. Due to the extremely strong *cis-* effect in F1s, we define them as “super *cis-*” genes (Fig 2A). As previously reported in other animal and plant species [2–5], we also discovered a substantial fraction of genes with significant *cis-* effects (43%), as well as genes with *trans-* effects (19%), suggesting that both play a role in expression divergence between the species (Fig 2B).

### Allele-Specific DNA Methylome Profiling in Reciprocal F1 Hybrids Reveals High Fidelity *Cis*-Regulation of Methylation in *Nasonia*

In mammals, DNA methylation is “reset” each generation during gametogenesis through a process called epigenetic reprogramming [49]. However, the patterns of DNA methylation maintenance and reprogramming are not known in insects. We can use F1 hybrids and the allele-specific differences in methylation between the two species to determine how methylation is maintained from parent to offspring. In particular, if DNA methylation changes during species divergence are purely due to changes in *cis*-regulatory sequences, then the F1 allele-specific methylation (ASM) will resemble the parental methylation status (the diagonal line in S1 Fig). If the methylation changes are due to *trans* factors only (e.g., methylation status is remodeled each generation), then F1 ASM will be ~50% on both V and G alleles with no interspecific differences (the horizontal line in S1 Fig). Our findings show that the allele-specific methylation pattern from the parent is maintained into adult F1 hybrid offspring with high fidelity, indicating a strong *cis*-regulation of DNA methylation and absence of remodeling between the embryo and F1 adult stages.

To characterize whether DNA methylation divergence between Nv and Ng is due to changes in *cis*-regulatory elements or *trans*-regulatory factors, we quantified ASM at the single CpG level with high-coverage whole-genome bisulfite sequencing (WGBS-seq) in whole adult samples of both reciprocal F1 hybrids. In our previous study, CpG methylation percentages were estimated by the proportion of unconverted Cs in Nv and Ng. We found that ~1.5% of the CpGs in the *Nasonia* genome are methylated [20]. Among the 6 million covered CpG sites (>10X read depth) in both species, 6,987 (8% of all ~90,000 methylated CpGs) are significantly differentially methylated (DM-CpGs) between Nv and Ng in both female and male comparisons (FDR<0.05, Fisher’s Exact Test). In the reciprocal F1 hybrids, we estimated total methylation percentage and allelic methylation for V and G alleles separately, using WGBS-seq reads containing informative SNPs to infer the parental origin of each allele (S3 Fig). Parental and F1 methylation percentages for nine selected genes were validated using PyroMark assays in both parental species and F1 hybrids from independent biological replicates, and all were confirmed (S3 and S4 Figs).

**Fig 3:**
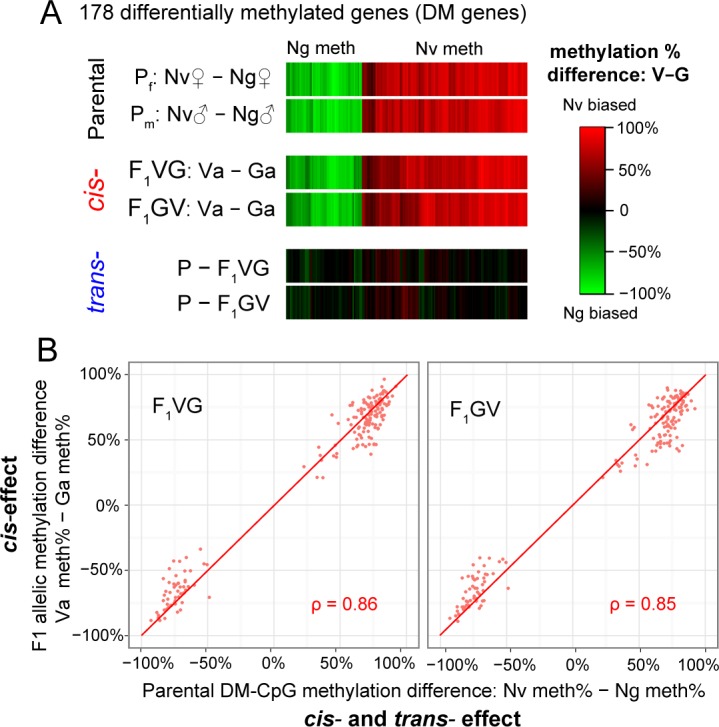
Differentially methylated genes between *N*. *vitripennis* and *N*. *giraulti* retain their parental methylation status into F1 adults. (A) Heat map showing that the 178 differentially methylated (DM) genes between *N*. *vitripennis* (Nv) and *N*. *giraulti* (Ng) also display strong allele-specific *cis*- regulation in both reciprocal F1 crosses (red: methylated in Nv; green: methylated in Ng). The top panel shows the parental methylation divergence for DM genes in females and males, quantified by Nv minus Ng methylation percentage in two sexes. The middle panel shows the *cis-*effect of DNA methylation in both reciprocal F1s, quantified by V allele (Va) methylation percentage minus the G allele (Ga) methylation percentage in F_1_VG and F_1_GV, respectively. Plotted in the bottom panel is the *trans*-effect, calculated as the total parental methylation divergence minus the *cis*-effect. The parental DNA methylation difference for DM genes could be explained by the *cis*-effect alone, with close to zero *trans*-effect in both reciprocal crosses. (B) Plotted on the *x*-axis are the parental methylation divergences averaged across differentially methylated CpGs (DM-CpGs) for each DM gene, measured by Nv minus Ng methylation percentage. On the *y*-axis are the F1 allelic methylation differences in F_1_VG (*left panel*) and F_1_GV (*right panel*), measured by *vitripennis* allele (Va) methylation percentage minus *giraulti* allele (Ga) methylation percentage in F1s. Spearman's rank correlation coefficients (ρ) are in the bottom right corner. Data presented in this figure can be found at http://dx.doi.org/10.5061/dryad.qf2t8.

In the *Nasonia* genome, the majority of methylated CpGs are clustered in coding exons [19]. Among the 6,987 DM-CpGs between Nv and Ng, 4,364 were covered with ≥8X for both the V and G alleles in each of the two reciprocal F1s (total coverage ≥16 in both F1s), allowing accurate estimation of allelic methylation percentages. Of these DM-CpGs, 2,461 are located in exons of 891 coding genes, and we define genes containing ≥4 DM-CpGs as differentially methylated genes (DM genes) between the two parental species Nv and Ng. There are 178 of these DM genes in the *Nasonia* genome (Fig 3A), and in all cases, the direction of differential methylation was identical across DM-CpGs within each gene. The metric for methylation divergence between the two species for 178 DM genes was defined as the CpG methylation percentage in Nv minus the methylation percentage in Ng (Fig 3A). Using similar metrics as *cis-* versus *trans-*effects on gene expression divergence (Fig 2), we quantified the *cis-*effect in methylation divergence by the methylation difference on the V and G alleles, which is calculated as the V allele (Va) methylation percentage minus the G allele (Ga) methylation percentage in F1s. These figures ranged from -100% to +100% (Fig 3); 100% implies exclusive Nv allele-specific methylation in the F1, and -100% implies exclusive Ng-specific methylation. 0% means no methylation difference between the two parental alleles, indicating complete lack of a *cis*-effect. *trans-*effects are measured by total methylation divergence (parental methylation difference) minus the F1 *cis-*effect (Fig 3A). For the 178 DM genes, when the F1 allelic methylation difference is plotted against the parental methylation difference, all points (Fig 3B) fall along the diagonal line for both F1 crosses, indicating high correlation between parental and F1 allelic methylation status. In the heat map (Fig 3A), we observed no significant *trans-*effect, and the parental methylation divergence could be explained entirely by *cis-*effects in the F1 progeny. Note that this absence of *trans*-regulation in methylation divergence is in striking contrast to expression divergence (Fig 2 and S1 Fig).

### Differentially Methylated Genes (DM Genes) Are Clustered in the *Nasonia* Genome Near Pericentric Regions

**Fig 4:**
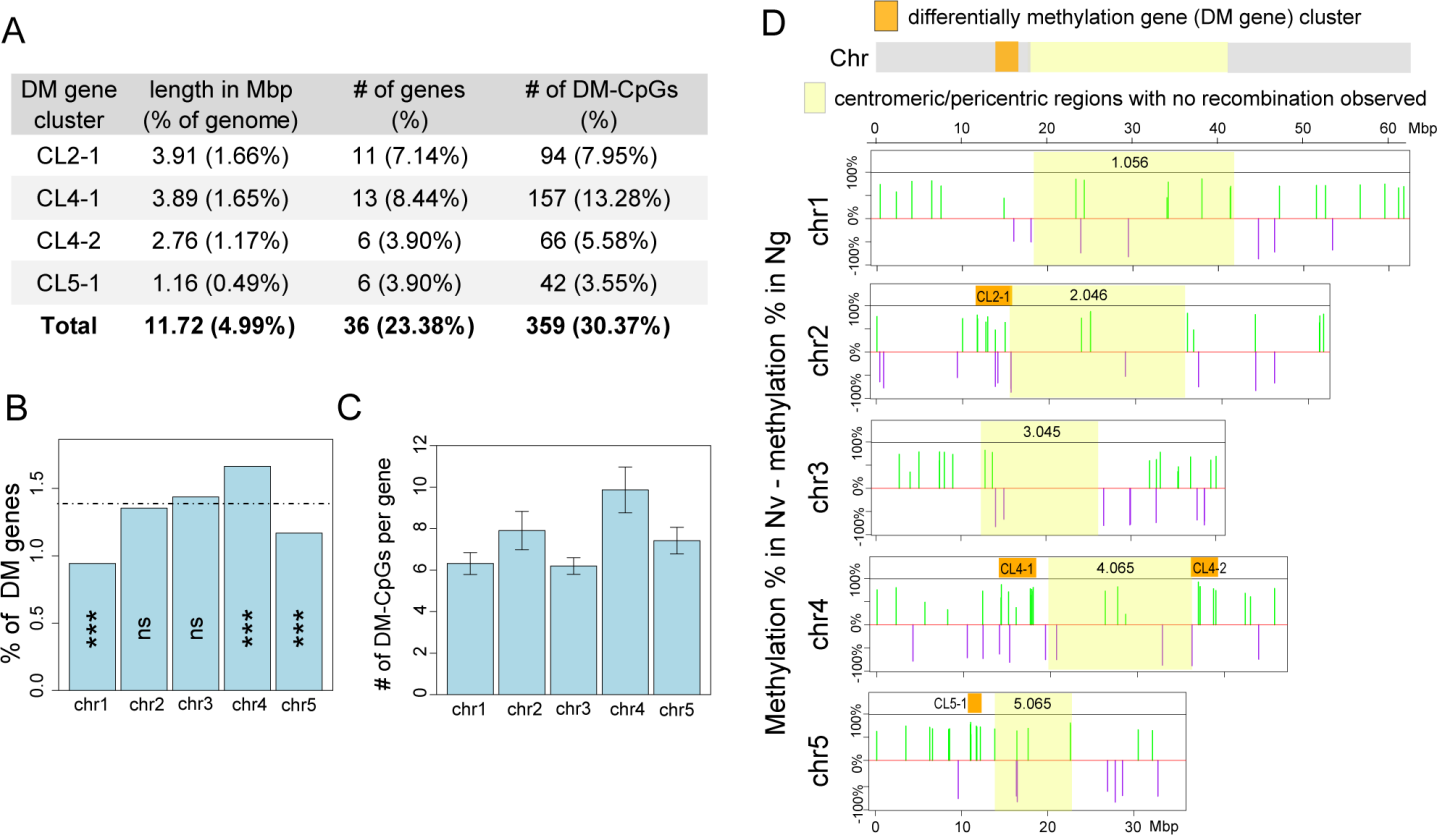
Genes that are differentially methylated (DM genes) between *N*. *vitripennis* and *N*. *giraulti* are clustered in the genome. **(A)** Summary of four DM gene clusters (CL2-1, CL4-1, CL4-2, and CL5-1) on *Nasonia* chr2, chr4, and chr5. The clusters’ physical length (in Mbp), number of DM genes in each cluster, number of differentially methylated CpGs (DM-CpGs), and their percentages in the entire genome are listed. **(B)** Barplot of percentage of DM genes on five *Nasonia* chromosomes. Expected percentage of genes for each chromosome under a random, uniform distribution is drawn as a dotted horizontal line. Statistical significance is calculated from permutation tests (ns: *p*-value > 0.05; *: *p*-value < 0.05; **: *p*-value < 0.01; ***: *p*-value < 0.001). **(C)** Barplot of average number of differentially methylated CpGs (DM-CpGs) per gene on five *Nasonia* chromosomes. **(D)** Plot of physical location and degree of DNA methylation divergence between Nv and Ng for 178 DM genes along five *Nasonia* chromosomes. The bar heights represent average methylation differences between Nv and Ng, calculated by Nv minus Ng methylation percentage. Nv methylated genes are labeled in green and Ng methylated genes are labeled in purple. The yellow-shaded regions are the centromeric/pericentromeric regions with extremely low recombination (no recombination observed in the population for generating the *Nasonia* linkage map in [50]). The four DM gene clusters are indicated by orange boxes. Data presented in this figure can be found at http://dx.doi.org/10.5061/dryad.qf2t8.

The genomic distribution of differentially methylated genes tend to fall into clusters, with one-fourth of the 178 genes that are differentially methylated between Nv and Ng occurring in four clusters (CL2-1, CL4-1, CL4-2, and CL5-1) near the pericentric regions of chr2, chr4, and chr5 (Fig 4A). These DM gene clusters only account for 5% of the genome in physical length, but they contain 23% of DM genes and 30% of DM-CpGs (Fig 4A). The number of DM genes is significantly enriched on chr4 (Fig 4B), which has two DM gene clusters. chr2, chr4, and chr5 also have the most DM-CpGs per gene (Fig 4C), which is largely driven by these DM clusters. The non-random distribution of DM genes (Fig 4D) might be caused by local differences in chromatin state between Nv and Ng. We speculate that these regions could play a role in phenotypic differences between the species and/or F2 hybrid breakdown.

### Correlation between Allele-Specific Expression (ASE) and Allele-Specific Methylation (ASM) Only Occurs in Extremely Differentially Methylated and Expressed Genes

**Fig 5:**
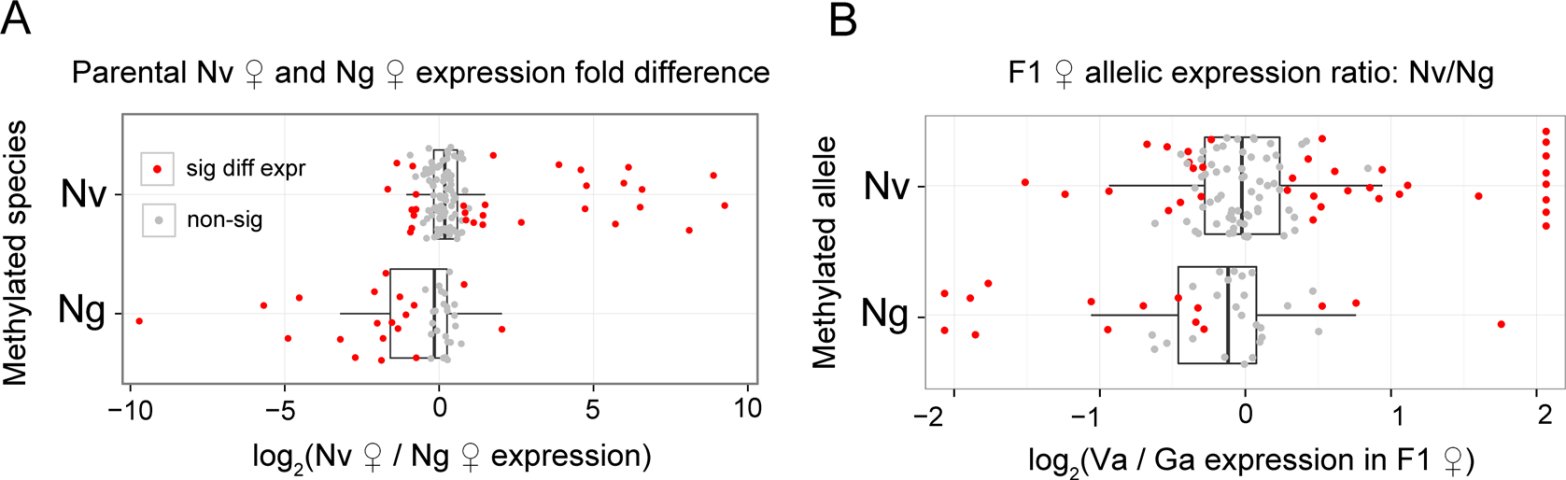
Differentially methylated genes between *Nasonia vitripennis* (Nv) and *Nasonia giraulti* (Ng) display a bias toward methylated alleles for both total and allelic expression levels. (A) Boxplot of parental Nv/Ng expression ratios for differentially methylated genes. Criteria are provided in materials and methods. Differentially expressed genes (FDR < 0.05) are shown in red. Among these genes, the methylated species show significantly higher expression compared to the species with the non-methylated allele. (B) Boxplot of F1 allele-specific expression ratios for differentially methylated genes between Nv and Ng, quantified by Nv allelic expression divided by Ng allelic expression in F1s (*x*-axis). Criteria are provided in materials and methods. Genes that are monoallelically expressed in Nv (Ng) were plotted at the right (left), because their log_2_ value is undefined. Genes with significant allelic expression bias (FDR < 0.01) are shown in red, and their methylation patterns are significantly biased toward the highly expressed alleles. Data presented in this figure can be found at http://dx.doi.org/10.5061/dryad.qf2t8.

In our previous study, we found that DNA methylation is positively correlated with expression level and expression breadth in *Nasonia vitripennis* [19]. However, the pattern alone does not establish causality between higher methylation and higher gene expression. To investigate whether differentially methylated genes between Nv and Ng display differential expression between Nv and Ng and/or allele-specific differential expression in F1 hybrids, we compared the parental expression ratios and F1 allelic expression ratios for Nv methylated versus Ng methylated genes (Fig 5). Among 178 such DM genes, 151 have detectable expression level in our RNA-seq data and an Nv-Ng methylation percentage difference greater than 50%. Of these, 49 (32.5%) are significantly differentially expressed between Nv and Ng females (DE genes). Only 11 of the 49 DE genes have higher expression in the non-methylated species and the fold difference between species is relatively small (average log_2_ fold change = 1.06; Fig 5A). In contrast, the number of DE genes that are highly expressed in the methylated species is significantly enriched (*p*-value = 0.0001, Chi-squared test; Fig 5A), with much larger fold difference between species (average log_2_ fold change = 3.54; *p*-value = 0.00035, Mann-Whitney U Test). Similar results were also observed when using the male total expression levels in Nv and Ng (S5 Fig). Therefore, we conclude that only about one-third of the DM genes show a significant between-species expression difference, and most are expressed highly in the more highly methylated species (Fig 5A). This suggests that DNA methylation might play a role in maintaining and/or strengthening the strong differential expression [19].

The result above would suggest that methylation accounts for the differential expression of these genes between the species, in only a subset of the DM genes. We therefore asked whether methylation differences between the species correlate with allele-specific methylation in F1 hybrids. Among 142 DM genes with high-quality informative SNPs to accurately quantify allele-specific expression, 54 display significant deviation from 50%:50% across all F1 replicates (FDR<0.01, one sample *t* test), indicating the presence of allelic imbalance (AI genes; Fig 5B). 69% (37) of these AI genes have an allelic expression bias toward the methylated allele in both hybrid F1s, which is a significantly higher proportion than expected by chance (*p*-value = 0.006, Chi-squared test). The average bias toward methylated allelic expression for these 37 genes is 75.2%, which is significantly higher than the bias toward non-methylated allele for the remaining 17 genes (62.7%, *p*-value = 0.019, Mann-Whitney U Test; Fig 5B). Just as was seen for total expression divergence, only about one-third of the DM genes show a significant allelic expression bias, the majority of which matched the methylated allele in the direction of allelic imbalance. There were 26 genes with significant allelic expression imbalance greater than 60% across all F1 samples, 12 of which have nearly mono-allelic expression of one parental allele in F1s (S6 Fig). These genes are all regulated in *cis* for expression divergence. Therefore we conclude that DNA methylation may contribute to *cis*-regulation, perhaps by serving to maintain and reinforce the *cis-*control of expression, but methylation is not a primary regulator of expression differences.

## Discussion

### Lack of Genomic Imprinting in Adult *Nasonia*

Imprinted genes are a subset of genes whose allelic expression depends on parent-of-origin. To date, in animals, genomic imprinting has been found in therian mammals, including marsupials [28]. Whether genomic imprinting is present in non-mammalian species is an intriguing evolutionary question. Many efforts have been made to search for imprinting and parent-of-origin expression in invertebrates, especially in eusocial insects. According to the kinship theory of genomic imprinting, differences in the relatedness of matrigenes and patrigenes can result in evolutionarily stable monoallelic expression of one parental allele when the patriline and matriline are subject to conflicting selective pressure [51]. The selective environment that results in patrigene-matrigene conflict occurs when offspring of a parent compete for resources and are differentially related through the patriline and matriline (e.g., due to multiple mating). This asymmetry in relatedness suggests that genomic imprinting could develop in species with social structure and social interactions among relatives, such as birds, mammals, and eusocial insects [51]. Using a candidate gene approach, a parent-of-origin expression pattern was found in two bumble bee genes, but it is still not clear whether this is due to genomic imprinting [46]. A recent study using F1 hybrids of African and European honey bees found significant parent-of-origin effects in gene expression, but most of the examples are asymmetric (monoallelic in one direction and biallelic in the reciprocal F1s) [47]. We checked these honey bee imprinting candidates with an ortholog in *Nasonia* and found that they do not differ in allelic expression ratios between the two reciprocal F1 crosses.

There are two genome-wide approaches to identify novel imprinted genes in mammals: quantification of parent-of-origin differential allelic expression (pDAE) to identify monoallelic expression directly from the transcripts, or identification of parent-of-origin dependent differentially methylated regions (pDMRs) in the genome. The latter asks whether the epigenetic regulatory mechanism of imprinting is in place, while the former asks whether the end result is differential parent-of-origin specific allelic expression. Our genome-wide transcriptome and methylome data from reciprocal F1 hybrids allow us to explore both pDAE and pDMRs. In contrast to the suggestive evidence of imprinting in bees, we did not find any imprinted genes, not even any weak or asymmetric pDAE in gene expression in F1s, although the SNP density between Nv and Ng is ideal to cover almost all expressed genes with informative SNPs.

If genomic imprinting exists in *Nasonia* and a similar pDMR mechanism is employed, we should observe paternal- or maternal-specific methylation at DMR regions in F1 hybrids, with 50% of overall DNA methylation in both F1 and parental females. Males carry only the maternal allele in a haploid state, and so an imprinted gene would be expected to display either 0% or 100% methylation. We found no such pDMRs in our WGBS-seq methylome dataset, which is consistent with a complete lack of pDAE. These findings and the stable inheritance of gene methylation status all suggest potential lack of epigenetic reprogramming of methylation in *Nasonia*. Interestingly, two recent studies in honey bee investigated DMRs at individual genes in F1s [52] or fertilized versus thelytokous embryos [53], and in both cases the DMRs were not associated with parent-of-origin. Instead, they showed *cis-*regulated sequence-driven allele-specific methylation [52,53], which is consistent with what we observed in *Nasonia*.

The lack of genomic imprinting in *Nasonia* is not surprising because kinship theory does not provide any reason to expect adult genomic imprinting in non-social wasps like *Nasonia*. These differences between non-social and eusocial hymenoptera in parent-of-origin differential allelic expression are consistent with the reduction of unequal relatedness between matrigenes and patrigenes in *Nasonia*, because it is inbred in nature and brother–sister mating is prevalent. However, since we used pooled adult whole body samples in this study, we have not ruled out the possibility of imprinting effects at a tissue-specific level or at other stages of development. For example, the larvae occur gregariously within parasitized hosts and there possibly is genetic conflict among *Nasonia* larvae, which compete within hosts for resources. However, the high level of inbreeding in these species [54] and relative infrequency of multiple mating [55] reduces the potential of patriline-matriline conflict and may explain why genomic imprinting appears absent in *Nasonia* relative to eusocial species.

### Presence of both *Cis*- and *Trans*-Regulatory Changes in Gene Expression Divergence and a Surprisingly Large Number of Super-*Cis* Genes

As we expected, after species divergence, DNA sequence changes that affect gene expression difference between Nv and Ng occurred both in *cis*-regulatory elements and *trans*-factors. The larger the expression divergence, the more *cis*-biased the effects are, suggesting that in *Nasonia* the primary changes are in *cis* and that *trans*-regulatory changes providing an additional level of modulation. This pattern is similar to findings in yeast [2] but not mice [5]. Interestingly, we discovered an unexpectedly large group of super *cis* genes (Fig 2C), which explains the largest expression divergence between the species (log_2_ fold difference > 4). After filtering out genes with signs of pseudogenization in one species and genes with indels between Nv and Ng, we are left with 50 super *cis* genes with Nv-biased expression and 11 with Ng-biased expression (Fig 2A). The skew in these numbers may be due to an ascertainment bias using the Nv reference genome. While gene ontology (GO) analysis did not reach statistical significance for any enriched functional categories, venom precursor, P450s for detoxification and transposase are among these super *cis* genes. Further experimental studies are needed to elucidate exactly how these genes are regulated in F1s.

### Strict *Cis*-Regulation of DNA Methylation Suggests Presence of Stable Determinants of CpG Methylation in *Cis*

We originally expected that DNA methylation divergence would be regulated in both *cis* and *trans*, just as is expression divergence. There was also a possibility of mis-regulation of DNA methylation in F1 hybrids, with methylation present at sites that are not methylated in either parental species, or lost in shared sites. In contrast, DNA methylation is highly conserved between Nv, Ng, and F1s at most methylated CpG sites. Surprisingly, in F1s, all differentially methylated genes are also differentially methylated between the two alleles, indicating that they “remember” the parental methylation status with nearly 100% fidelity. The finding suggests the presence of strong DNA sequence element(s) in *cis* guiding the DNA methylation machinery to target CpG methylation. Identification of this potential motif is a clear goal for future research.

An alternative hypothesis is that the stable inheritance of methylation status is maintained in *cis* with high fidelity by an epigenetic tag without any *cis-*element in the DNA sequence. Methylated CpGs could themselves serve as the *cis*-elements for methylation maintenance, but this would require a very high fidelity of maintenance and absence of methylation remodeling during early development. In mammals, DNMT1 is the maintenance enzyme and preferentially methylates hemimethylated DNA [56]. However, in insects, the enzymatic functions of DNMTs are actually not known but, rather, inferred from studies in vertebrates and plants. Furthermore, mammals and insects differ in the numbers and types of DNMTs. For example, there is an expansion of DNMT3s in mammals versus DNMT1s in *Nasonia*, while other insects show different patterns [18]. Therefore, these enzymes could have evolved novel roles for high fidelity maintenance of DNA methylation across generations, and their functional roles in insects need to be further investigated.

In mammals, there are two waves of de- and re-methylation called epigenetic reprogramming [49,57–61]. During gametogenesis in the primordial germ cells (PGCs), the biparental methylation marks are erased and then reestablished according to the parent sex (paternal imprints or maternal imprints), and this is how the parental specific differential methylation marks of imprinted genes are transmitted through generations. The reprogramming processes in male and female germ lines differ in timing, length and degree of de-methylation, with distinct methylome patterns in male and female gametes. After fertilization, there is another wave of global erasure and resetting of DNA methylation in embryos, but most primary pDMRs of imprinted genes are not affected in this round, and therefore maintain the differential methylation status. The lack of pDAE/pDMRs and stable inheritance of DNA methylation in *Nasonia* suggest potential lack of epigenetic reprogramming. However, with our current data we cannot rule out another possibility of extensive reprogramming in *Nasonia*. Formally, one could argue that the erasure of DNA methylation occurs, but methylation status is then restored with high fidelity. However, this seems unlikely because reprogramming would hardly be necessary under this scenario. If this type of erasure-restoring exists, the re-establishment of DNA methylation pattern could be regulated genetically by DNA motifs in *cis* to guide the erasure and resetting, or epigenetically through stable maintenance by DNMTs, small RNA pathways, or other unknown mechanisms.

Many cases of transgenerational epigenetic inheritance have been discovered in flowering plants, which is generally absent in mammals [62]. Unlike insect methylation, which is mainly located in coding sequences, in plants stable transgenerational epigenetic inheritance of DNA methylation targets and silences transposable elements and repeats [63], through an RNA-directed DNA methylation (RdDM) mechanism involving Pol IV and Pol V [64,65]. Pol IV and Pol V are not found in animals, so we do not think the stable inheritance of DNA methylation in *Nasonia* is regulated epigenetically by the RdDM mechanism as in plants. Due to the limited knowledge of insect DNA methylation regulation, whether germ line epigenetic reprogramming exists and how stable inheritance of DNA methylation is achieved are still open questions for future investigations.

### Expression-Methylation and ASE-ASM Correlations Are Generally Weak

A link between allele-specific methylation and increased allelic expression in insects was first described in the ant species *Camponotus floridanus* and *Harpegnathos saltator* [11]. In *Nasonia*, we have shown that gene methylation is positively correlated with expression level, and methylated genes tend to have housekeeping functions and a broad expression breadth [19]. Furthermore, methylation status is conserved across long evolutionary time scales, and methylated genes have evolved more slowly. To study the effect of DNA methylation on total expression differences between species and allele-specific expression in F1s, we quantified the Nv/Ng total and allelic expression ratios and found that overall DM genes do not consistently show higher expression between the species or allele-specific expression in F1 hybrids. Furthermore, DE gene expression between the species and in hybrids is not strongly correlated with methylation status. However, for the ~30% DM genes that display significant between-species expression differences and F1 allelic expression bias, the methylated allele is more highly expressed in hybrids most of the time. These findings suggest that methylation does not strongly predict gene expression level, but may be a contributing factor. After expression differences are established, we speculate that DNA methylation may provide a further signal to promote expression of the more highly expressed allele, as well as to stabilize the expression level (e.g., reduce noise in expression level) across development and cell types for “housekeeping” genes [19]. We further note that methylation is associated with the initiation site of the protein coding region of transcribed genes, and therefore may provide a signal for an epigenetic tag of RNA for the start codon, thus promoting more efficient translation [19].

### Clustering of Differentially Methylated Genes May Correlate with Local Chromatin State and Serve as an Epigenetic Component for Speciation

Differentially methylated genes are not randomly distributed. Pericentric regions of chr2, chr4, and chr5 are enriched for DM genes. The number of DM-CpGs and degree of parental methylation differences are also elevated in these regions. We speculate that the organization of DM genes might be associated with local chromatin state. The regional epigenetic differences could contribute to phenotypic differences between species. In addition, the clustered epigenetic divergences might reinforce the reproductive barriers after species divergence. In *Drosophila*, the differences in chromatin state across flies with different sex chromosome configurations were also found to cluster near the pericentromeric regions [66]. Proximity to centromeric heterochromatin may impose a regional effect on chromatin state, driving the clustering seen here and in our *Nasonia* data. One possibility is that DNA methylation serves to increase accessibility of DNA around functional genes within heterochromatin, which may explain these clusters. Due to the small number of DM genes and the fact that some of them lack an annotated function, functional enrichment analysis did not identify any significant category, but there are a list of genes with interesting epigenetic and splicing functions, such as chromodomain Y protein, factors involved in splicing, polyadenylation factors, RNA binding proteins, helicases, and tRNA methyltransferases. These are interesting genes for follow-up studies to investigate the biological significance of species differences in DM genes.

## Materials and Methods

### RNA-seq and WGBS-seq Experiments in Reciprocal F1 Hybrids of *Nasonia vitripennis* and *Nasonia giraulti*

Total RNA samples were extracted from a pool of ten 24 h post-eclosion whole adult samples from reciprocal F1 crosses (F_1_VG and F_1_GV) of *N*. *vitripennis* (strain AsymCX) and *N*. *giraulti* (strain R16A), using Qiagen RNeasy Plus mini kit (Qiagen, CA). RNA-seq libraries were prepared with 2 μg of input total RNA using TruSeq RNA Sample Preparation Kits v2 (Illumina Inc., CA), and sequenced on an Illumina HiSeq2000 instrument following standard Illumina RNA-seq protocols. Three independent biological replicates were performed for each cross, with an average of 60.7 million 51-bp single-end short reads per replicate.

Genomic DNAs were extracted from a pool of 50 24 h adult samples from both F_1_VG and F_1_GV using the DNeasy Blood & Tissue Kit (Qiagen, CA). WGBS-seq libraries were made with 20 μg of input genomic DNA according to a modified Illumina protocol described in our previous studies [19,20], and sequenced on Illumina HiSeq2000 instrument. The bisulfite conversion rate is greater than 99.9%, estimated from non-methylated lambda DNA (Cat No. D1521, Promega, WI), which went through all the library preparation and sequencing process with the *Nasonia* samples. Greater than 45X average haploid genome coverage is achieved for both samples.

### RNA-seq Alignments, Gene Expression and Allelic Expression Analysis

Initial QC of Illumina reads was performed with FastQC software [67] and adapter trimming was done by Trimmomatic [68], before they were aligned to *N*. *vitripennis* and *N*. *giraulti* reference genomes (v1.0) [69] using TopHat v2.0 [70]. More than 90% of reads were uniquely mapped to the reference genome except F_1_GV replicate 2, and it was excluded from the analysis. Read counts were summarized for each OGS2 (official gene set 2) genes [19,71] using three software packages: Cufflinks v2.1.0 [72], HTSeq [73], and BEDTools [74]. We manually corrected 23 inconsistencies among these three methods by checking the alignments. Normalization, estimation of expression levels and calling of differentially expressed genes were performed using the edgeR package in Bioconductor [75,76]. 16,400 expressed genes, which had an average expression level of FPKM > 1 across three biological replicates in at least one sample, were included in the analysis. Differentially expressed genes between the two sexes and the two species were detected by edgeR using 5% FDR (false discovery rate, *q*-value < 0.05). Using all mapped reads (including reads mapped to multiple places in the genome) and uniquely mapped reads only gave almost identical results and the data used in the figures are uniquely mapped reads only. The RNA-seq data in two parental species Nv female, Nv male, Ng female, and Ng male is from our previous work with accession numbers GSE43422 and GSE61156 [19,20]. The exonic SNP density between Nv and Ng allows us to estimate allele-specific expression for nearly all expressed genes in reciprocal F1s. To achieve accurate estimation of allele-specific expression in F1 RNA-seq data, we restricted the analysis among the 301 k SNPs called from Nv and Ng genomic DNA-seq data [20]. Informative V-G exonic SNPs with read depth 30 or more were included in the final analysis. Allelic expression was estimated for 9,869 genes with at least two independent informative SNPs and 109 genes with only one informative SNP and greater than 100X coverage. To detect significant parent-of-origin effect, we performed Fisher’s exact test using counts from the two parental alleles in both F1s in each replicates [48]. Replicated G-test of independence was performed to test whether the parent-of-origin effect is homogenous across all replicates [77]. The replicates were found to have greater variance than expected by a simple binomial, and so we applied fits to a beta-binomial (R package vgam) to accommodate this over-dispersion and test significance of reciprocal cross differences.

### Inference of *Cis-* and *Trans-*Effects on Expression Divergence in F1 RNA-seq Data

Significant *cis*- and *trans*-effect on gene expression divergence was accessed using a method described in [3]. *Cis*-effect is quantified as log_2_(V/G allelic expression in F1s) and the *trans-*effect is calculated as the expression divergence (log_2_ Nv/Ng total expression in parental species) subtract the *cis-*effect. Since the two reciprocal F1 hybrids do not have any significant parent-of-origin effect (Fig 1), we combined the replicates from both F1s in this analysis.

### WGBS-seq Alignments, Analysis of CpG Methylation and Estimation of Allele-Specific Methylation in Reciprocal F1 Hybrids

After QC, adapter trimming and quality filtering, 130 million 101 bp single-end WGBS-seq reads were left for F_1_VG and 170 million for F_1_GV. These reads were aligned to both Watson and Crick strands of the converted *N*. *vitripennis* and *N*. *giraulti* reference genome (v1.0) using BWA [78], allowing up to four mismatches for Nv and six mismatches for Ng genome. A detailed protocol can be found in our previous publication [19]. 85% of all the reads were aligned to the converted genomes in both species and only 60% of those that uniquely mapped to the genome were kept for the methylation analysis. To infer allele-specific methylation, we assigned the parental transmission direction for the WGBS-seq reads with an informative V-G SNP or a G-specific indel in them. Among the uniquely mapped reads, 36% do not contain any V-G fixed differences or indels and they were excluded from the allele-specific methylation analysis; 36% came from the Nv parent and 28% came from the Ng parent. The unequal number of Nv and Ng parental reads is due to reference genome mapping bias, which was corrected subsequently. To achieve accurate estimation of allele-specific methylation, we restricted our analysis on the CpGs with at least 8X Nv allele coverage and 8X Ng allele coverage (total coverage > 16X). Methylation percentages for the Nv and Ng alleles were estimated separately in both reciprocal F1s. The total methylation percentages were also calculated. Aligned BAM files were viewed in the IGV browser [79,80].

### Inference of *Cis-* and *Trans-*Effects on Expression Divergence in F1 RNA-seq Data

Parental methylome data for Nv female, Nv male, Ng female, and Ng male are from our previous research with accession numbers GSE43423 and GSE61158 [19,20]. CpGs that were differentially methylated between the two parental species (DM-CpGs) were called using the method described here [20]. 6,987 significant DM-CpGs between Nv and Ng are shared in both female and male comparisons (*q* < 0.05, Fisher’s Exact Test). Among these, 4,364 have at least 8X coverage for both V allele and G allele in each of the two reciprocal F1s (total coverage ≥16). Of parental DM-CpGs covered in F1s, 2,461 are in the transcript regions of 891 OGS2 genes. One hundred and seventy-eight genes with four or more DM-CpGs are defined as differentially methylated genes (DM genes) between Nv and Ng. To quantify the degree of *cis*-effect of DNA methylation divergence, we calculated the differences between the V allelic methylation and G allelic methylation in F1s, ranging from -100% to +100%. +100% means 100% methylation at V allele and 0% G allelic methylation in F1s, suggesting strong *cis*-effect toward the V allele. Likewise, -100% suggests G allele-specific methylation in F1s. If there is complete lack of *cis*-effect and no methylation difference between the two parental alleles, the metric will be 0%. The statistical significance is accessed using *t* test across all covered DM-CpG sites.

### Validation of CpG Methylation Percentages for Selected Differentially Methylated Genes in Parental and Both Reciprocal F1 Samples Using PyroMark Assays

Genomic DNA samples were extracted from a pool of 50 24 h post-eclosion adult individuals in Nv male, Nv female, Ng male, Ng female, F_1_VG and F_1_GV using the Qiagen DNeasy Blood and Tissue Kit (Qiagen, CA, Cat No. 69504). Two independent biological replicates collected from different batches were done for each strain/sex and of each, two technical replicates were performed for PyroMark. For bisulfite conversion, 2 μg of genomic DNA were used with Qiagen EpiTect Bisulfite Kit (Qiagen, CA). For each gene, PyroMark primers were designed to overlap only with invariant positions between Nv and Ng using PyroMark Assay Design Software Version 2.0.1.15 (Qiagen, CA), and they were tested to work for both species. Target CpG regions were selected in 13 differentially methylated genes between Nv and Ng, and 9 of them have strong signal for accurate quantification of CpG methylation percentages (S4 Fig). PCR products were prepared, run, and analyzed on the PSQ 96MA Pyrosequencer (Qiagen, CA) with PyroMark CpG software 1.0.11.

## Supporting Information

S1 Fig A depiction of *cis*- versus *trans*-regulatory divergence from hybrid F1s of two closely related species. (**A**) Suppose two closely related species (species A and B) are evolved from a single ancestral species. After divergence, some orthologous genes are differentially expressed between the two species. The gene expression divergence could be attributed to species-specific DNA sequence changes in *cis-* element (C) and/or *trans-* factors (D), which could be quantified using interspecific F1 hybrids. (**B**) A diagram of a hypothetical scatterplot of the relative F1 allelic expression ratio of the two alleles (aA/aB on *y*-axis) against the relative total expression in the two parental species (SA/SB on *x*-axis). (**C**) Suppose a gene has 2-fold higher expression in species A (green on the top left) than species B (purple on the top right). The presence of *cis-*regulatory changes in promoter/enhancer regions (green versus purple boxes) can alter expression regulation. Under pure *cis*-regulatory divergence, the effect will be allele-specific in F1s. If we plot the relative F1 allelic expression ratio (aA/aB) against the expression ratio in the two parental species (SA/SB), they will be in proportion on the diagonal line (red line in B). (**D**) Under pure *trans-*regulatory divergence, the *trans-*factors from the two species regulate both parental alleles in F1 hybrids. Therefore there will be no allelic imbalance and 50%:50% allelic expression is expected in F1s (blue line in B).
(PDF)

S2 Fig Total expression correlation between whole adult samples from reciprocal crosses F_1_VG and F_1_GV. Plotted on the *x*-axis is the total expression level for 8,622 genes in F_1_VG progeny RNA-seq data quantified by log_2_(FPKM). Plotted on the *y*-axis is the total expression level in the reciprocal F_1_GV progeny. Significant differentially expressed genes are labeled in red and non-significant genes in black. Data presented in this figure can be found at http://dx.doi.org/10.5061/dryad.qf2t8.
(PDF)

S3 Fig Estimation and validation of allele-specific methylation and detection of *cis-*regulation of DNA methylation for *Ank2* gene. (A) Plot of the exon model, translation start site and CpG methylation profile for *Ank2* gene in Nv female, Nv male, Ng female, Ng male, F_1_VG, and F_1_GV (from top to bottom). A vertical bar is drawn for each covered CpG at its position in the gene, color-coded by the methylation percentage in proportion to the bar height (blue: proportion of methylated Cs; red: proportion of un-methylated Cs that are converted to Ts). For F1 samples, the methylation profiles for the V allele (Va) and G allele (Ga) are plotted separately. Among 112 CpGs covered with a read depth of 10 or more in all parental samples, 20 (DM-CpGs) displayed significantly different methylation between Nv and Ng. The average methylation percentages for these 20 DM-CpGs are labeled on the right of each panel. (B) IGV browser screenshot of WGBS-seq alignments for a 57 bp region in Nasvi2EG017594 on SCAFFOLD77 for Nv female (*top left*), Ng male (*bottom left*), and F_1_VG (*right*, Nv and Ng alleles sorted in separate panels), showing two DM-CpG sites and their methylation percentages (blue pointing arrows on the top of each panel), as well as two informative SNP positions between Nv and Ng (red arrow at the bottom). The two CpGs are only methylated in the Nv mother but not the Ng father. Allelic methylation analysis in F_1_VG showed methylated CpGs are exclusively on the Nv allele, resembling the paternal status. (C) Validation of the methylation percentages in Nv, Ng, and F1 by PyroMark assay. Raw pyrograms are shown for the methylation quantification of the four DM-CpG sites shown in (A). The assays were performed with two technical replicates using the same primer set and in the same batch for all six samples. Data presented in this figure could be found at http://dx.doi.org/10.5061/dryad.qf2t8.
(PDF)

S4 Fig Validation of methylation percentages for nine differentially methylated genes between *Nasonia vitripennis* (Nv) and *Nasonia giraulti* (Ng) using the PyroMark assay in independent biological replicates. Plotted in each bar plot are CpG methylation percentages estimated from whole-genome bisulfite sequencing data (left six bars) and single gene PyroMark validation (right six bars). For each assay, from left to right are the methylation percentages in Nv female, Nv male, Ng female, Ng male, F_1_VG, and F_1_GV. Nv methylation and V allelic methylation in F1s are labeled in green, and Ng methylation and G allelic methylation in F1s are labeled in purple. The PyroMark cannot distinguish allelic methylation, therefore the F1 total methylation is labeled in gray. Data presented in this figure can be found at http://dx.doi.org/10.5061/dryad.qf2t8.
(PDF)

S5 Fig Boxplot of parental Nv/Ng male expression difference for differentially methylated gene between *Nasonia vitripennis* (Nv) and *Nasonia giraulti* (Ng). Differentially expressed genes (FDR < 0.05) are shown in red. Among these genes, methylated species show significantly higher expression between species. Data presented in this figure can be found at http://dx.doi.org/10.5061/dryad.qf2t8.
(PDF)

S6 Fig Methylation and allelic expression profile for 26 differentially genes with allelic expression bias. Plot of parental/F1 methylation and F1 differentially allelic expression profile for 26 differentially methylated genes with 60% or more allelic expression bias. For DNA methylation, the upper panel is the methylation percentage in Nv or the V allele methylation percentage in F1s, and the lower panel is the methylation percentage in Ng or the G allele methylation percentage in F1s. For each gene, the first bar from the left is the Nv (green) and Ng (purple) methylation percentages in parental species. The second and third bars are the V allele (green) and G allele (purple) methylation percentages in F_1_VG and F_1_GV, respectively. The points with error bars on the right *y*-axis are differential allelic expression in F1s. Data presented in this figure can be found at http://dx.doi.org/10.5061/dryad.qf2t8.
(PDF)

S1 Table List of super *cis-* genes and their functional annotation. (PDF)

## Abbreviations

ASE: allele-specific expression
ASM: allele-specific methylation
DE genes: differentially expressed genes
DM genes: differentially methylated genes
DM-CpGs: differentially methylated CpG sites
DMR: differentially methylated region
DNMT: DNA methyltransferases
FDR: false discovery rate
FPKM: fragments per kilobase of transcript per million mapped reads
ICRs: imprinting control regions
iQTLs: imprinting quantitative trait loci
pDAE: parent-of-origin differential allelic expression
pDMRs: parent-of-origin dependent differentially methylated regions
PGCs: primordial germ cells
RdDM: RNA-directed DNA methylation
WGBS-seq: whole-genome bisulfite sequencing
