# Supplementary material for: Allele-Specific Transcriptome and Methylome Analysis Reveals Stable Inheritance and Cis-Regulation of DNA Methylation in Nasonia
Source: PLoS Biol. 2016 Jul 5;14(7):e1002500. doi: 10.1371/journal.pbio.1002500 (PMC4933354; doi:10.1371/journal.pbio.1002500)
Supplement: S1 Fig — (A) Suppose two closely related species (species A and B) are evolved from a single ancestral species. After divergence, some orthologous genes are differentially expressed between the two species. The gene expression divergence could be attributed to species-specific DNA sequence changes in cis- element (C) and/or trans- factors (D), which could be quantified using interspecific F1 hybrids. (B) A diagram of a hypothetical scatterplot of the relative F1 allelic expression ratio of the two alleles (aA/aB on y-axis) against the relative total expression in the two parental species (SA/SB on x-axis). (C) Suppose a gene has 2-fold higher expression in species A (green on the top left) than species B (purple on the top right). The presence of cis-regulatory changes in promoter/enhancer regions (green versus purple boxes) can alter expression regulation. Under pure cis-regulatory divergence, the effect will be allele-specific in F1s. If we plot the relative F1 allelic expression ratio (aA/aB) against the expression ratio in the two parental species (SA/SB), they will be in proportion on the diagonal line (red line in B). (D) Under pure trans-regulatory divergence, the trans-factors from the two species regulate both parental alleles in F1 hybrids. Therefore there will be no allelic imbalance and 50%:50% allelic expression is expected in F1s (blue line in B). (PDF) [file pbio.1002500.s001.pdf]

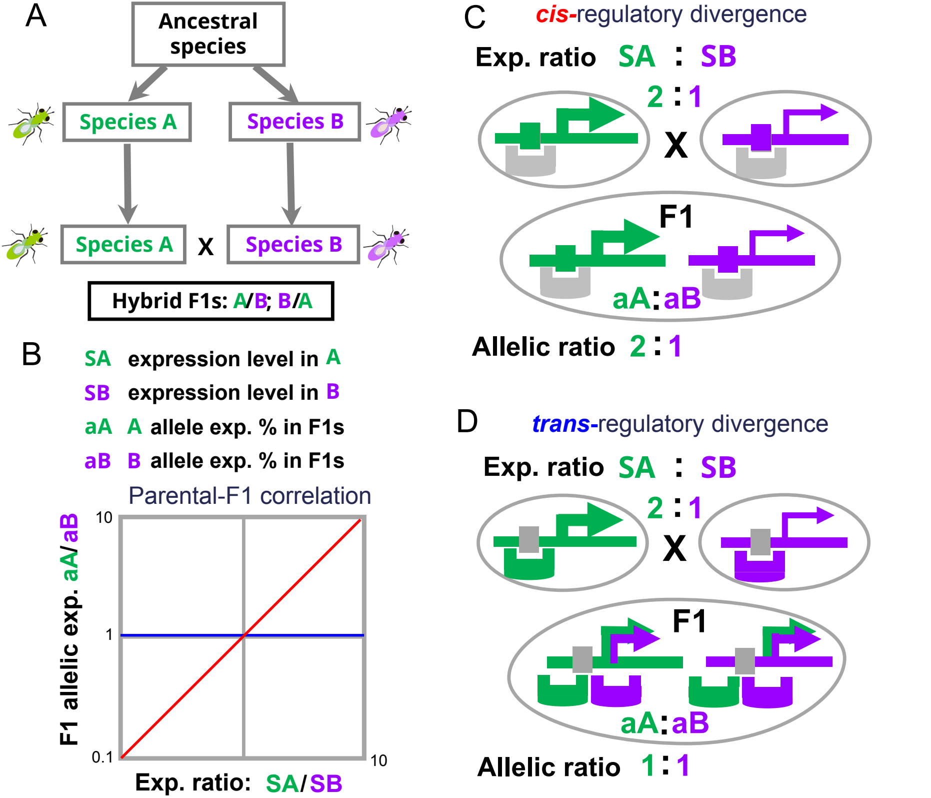
A
Ancestral species
Species A
Species B
Species A
X
Species B
Hybrid F1s: A/B; B/A
B
SA expression level in A
SB expression level in B
aA A allele exp. % in F1s
aB B allele exp. % in F1s
Parental-F1 correlation
F1 allelic exp. aA/aB
10
1
0.1
10
Exp. ratio: SA/SB
C
cis-regulatory divergence
Exp. ratio SA : SB
2 : 1
X
F1
aA:aB
Allelic ratio 2 : 1
D
trans-regulatory divergence
Exp. ratio SA : SB
2 : 1
X
F1
aA:aB
Allelic ratio 1 : 1
