# Supplementary material for: Allele-Specific Transcriptome and Methylome Analysis Reveals Stable Inheritance and Cis-Regulation of DNA Methylation in Nasonia
Source: PLoS Biol. 2016 Jul 5;14(7):e1002500. doi: 10.1371/journal.pbio.1002500 (PMC4933354; doi:10.1371/journal.pbio.1002500)
Supplement: S2 Fig — Plotted on the x-axis is the total expression level for 8,622 genes in F1VG progeny RNA-seq data quantified by log2(FPKM). Plotted on the y-axis is the total expression level in the reciprocal F1GV progeny. Significant differentially expressed genes are labeled in red and non-significant genes in black. Data presented in this figure can be found at http://dx.doi.org/10.5061/dryad.qf2t8. (PDF) [file pbio.1002500.s002.pdf]

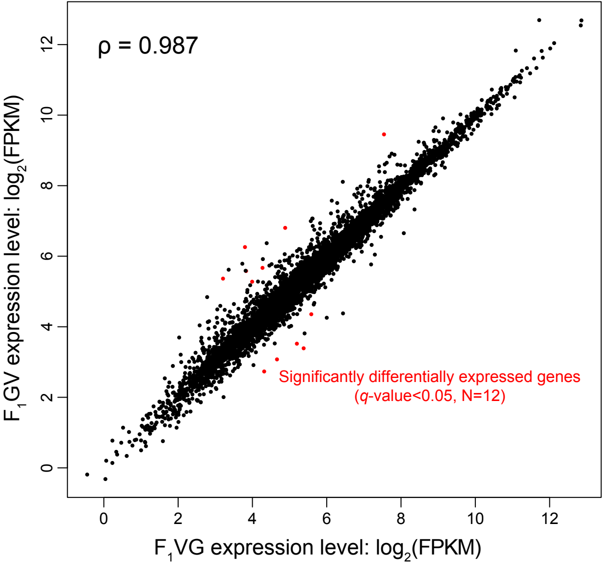
ρ = 0.987
Significantly differentially expressed genes
(q-value<0.05, N=12)
0
2
4
6
8
10
12
F1VG expression level: log2(FPKM)
F1GV expression level: log2(FPKM)
