# Supplementary figures and images for: Allele-Specific Transcriptome and Methylome Analysis Reveals Stable Inheritance and Cis-Regulation of DNA Methylation in Nasonia
Source: PLoS Biol. 2016 Jul 5;14(7):e1002500. doi: 10.1371/journal.pbio.1002500 (PMC4933354; doi:10.1371/journal.pbio.1002500)

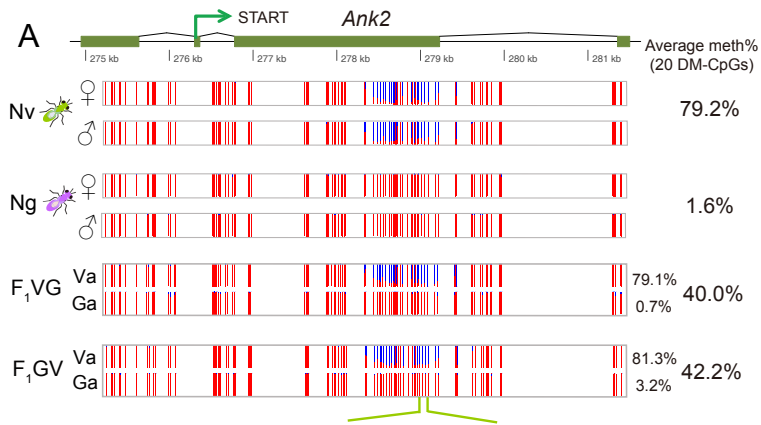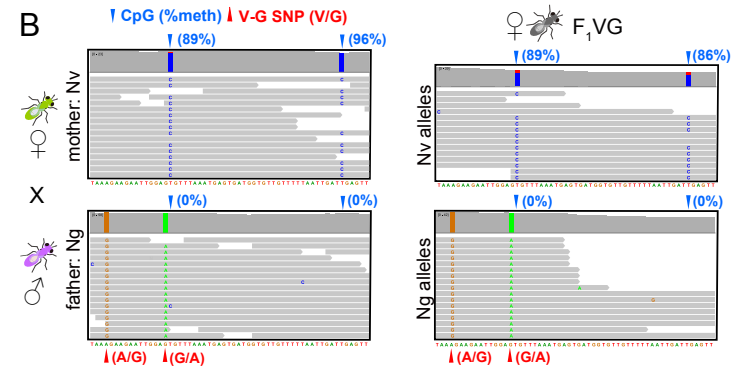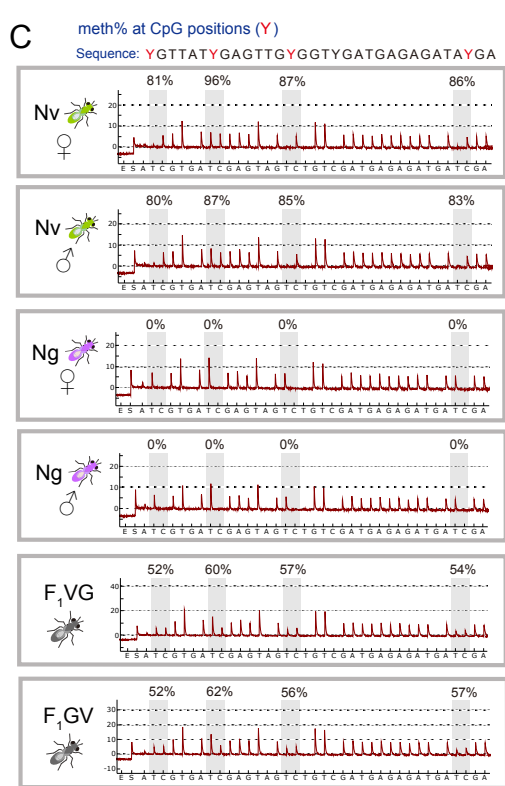

Supplement: S3 Fig — (A) Plot of the exon model, translation start site and CpG methylation profile for Ank2 gene in Nv female, Nv male, Ng female, Ng male, F1VG, and F1GV (from top to bottom). A vertical bar is drawn for each covered CpG at its position in the gene, color-coded by the methylation percentage in proportion to the bar height (blue: proportion of methylated Cs; red: proportion of un-methylated Cs that are converted to Ts). For F1 samples, the methylation profiles for the V allele (Va) and G allele (Ga) are plotted separately. Among 112 CpGs covered with a read depth of 10 or more in all parental samples, 20 (DM-CpGs) displayed significantly different methylation between Nv and Ng. The average methylation percentages for these 20 DM-CpGs are labeled on the right of each panel. (B) IGV browser screenshot of WGBS-seq alignments for a 57 bp region in Nasvi2EG017594 on SCAFFOLD77 for Nv female (top left), Ng male (bottom left), and F1VG (right, Nv and Ng alleles sorted in separate panels), showing two DM-CpG sites and their methylation percentages (blue pointing arrows on the top of each panel), as well as two informative SNP positions between Nv and Ng (red arrow at the bottom). The two CpGs are only methylated in the Nv mother but not the Ng father. Allelic methylation analysis in F1VG showed methylated CpGs are exclusively on the Nv allele, resembling the paternal status. (C) Validation of the methylation percentages in Nv, Ng, and F1 by PyroMark assay. Raw pyrograms are shown for the methylation quantification of the four DM-CpG sites shown in (A). The assays were performed with two technical replicates using the same primer set and in the same batch for all six samples. Data presented in this figure could be found at http://dx.doi.org/10.5061/dryad.qf2t8. (PDF) [file pbio.1002500.s003.pdf]

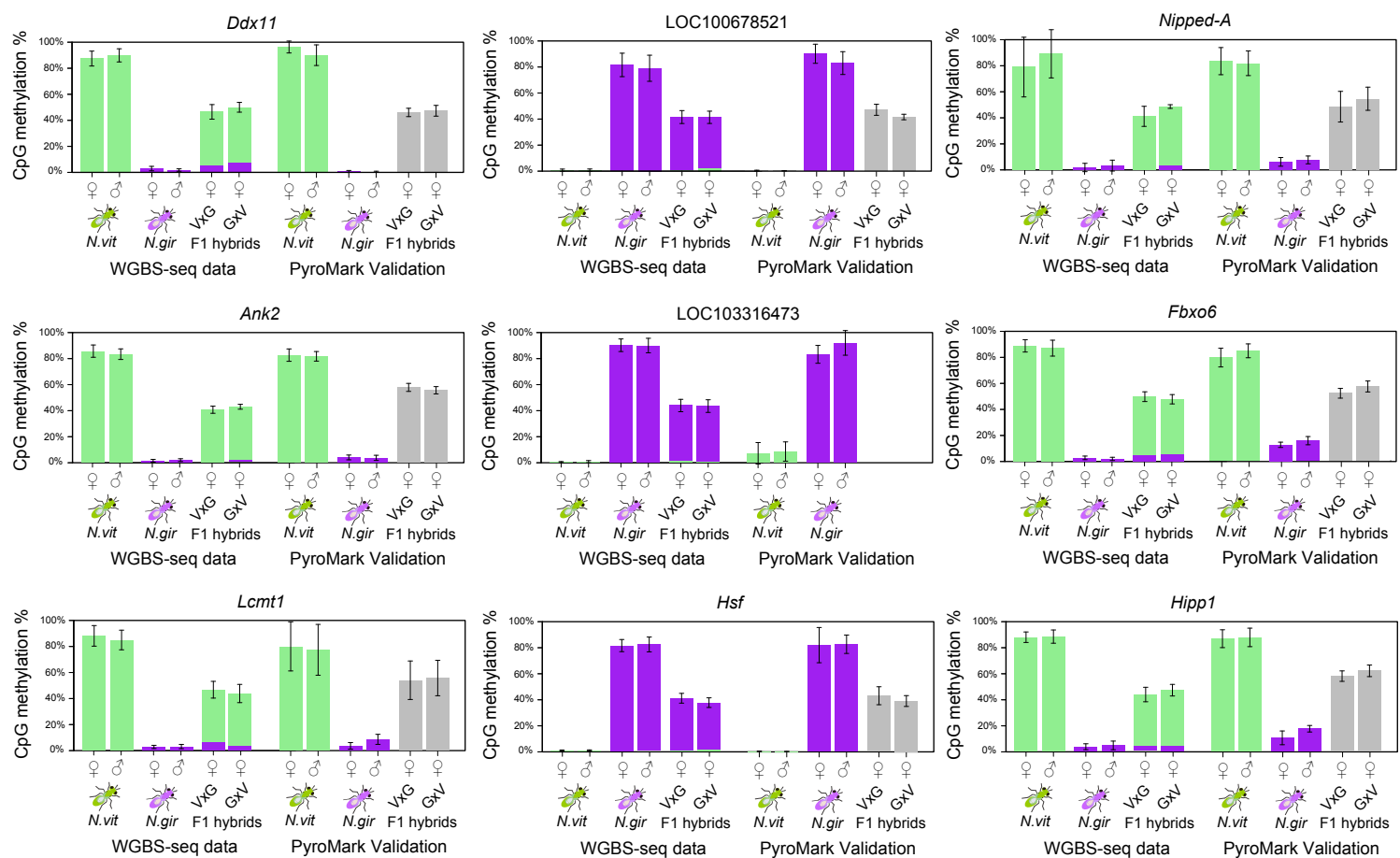

Supplement: S4 Fig — Plotted in each bar plot are CpG methylation percentages estimated from whole-genome bisulfite sequencing data (left six bars) and single gene PyroMark validation (right six bars). For each assay, from left to right are the methylation percentages in Nv female, Nv male, Ng female, Ng male, F1VG, and F1GV. Nv methylation and V allelic methylation in F1s are labeled in green, and Ng methylation and G allelic methylation in F1s are labeled in purple. The PyroMark cannot distinguish allelic methylation, therefore the F1 total methylation is labeled in gray. Data presented in this figure can be found at http://dx.doi.org/10.5061/dryad.qf2t8. (PDF) [file pbio.1002500.s004.pdf]

# Parental Nv ♂ and Ng ♂ expression fold difference

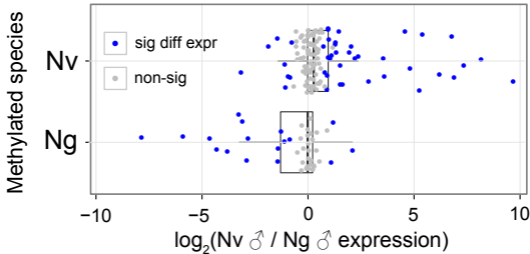

Supplement: S5 Fig — Differentially expressed genes (FDR < 0.05) are shown in red. Among these genes, methylated species show significantly higher expression between species. Data presented in this figure can be found at http://dx.doi.org/10.5061/dryad.qf2t8. (PDF) [file pbio.1002500.s005.pdf]

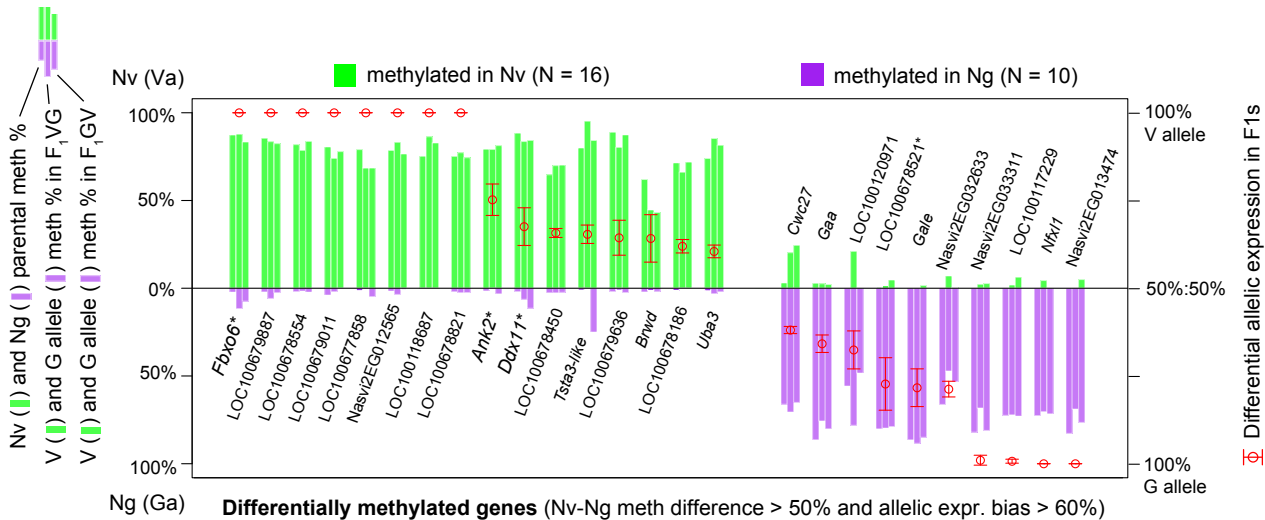

Supplement: S6 Fig — Plot of parental/F1 methylation and F1 differentially allelic expression profile for 26 differentially methylated genes with 60% or more allelic expression bias. For DNA methylation, the upper panel is the methylation percentage in Nv or the V allele methylation percentage in F1s, and the lower panel is the methylation percentage in Ng or the G allele methylation percentage in F1s. For each gene, the first bar from the left is the Nv (green) and Ng (purple) methylation percentages in parental species. The second and third bars are the V allele (green) and G allele (purple) methylation percentages in F1VG and F1GV, respectively. The points with error bars on the right y-axis are differential allelic expression in F1s. Data presented in this figure can be found at http://dx.doi.org/10.5061/dryad.qf2t8. (PDF) [file pbio.1002500.s006.pdf]
