# Supplementary material for: Allele-Specific Transcriptome and Methylome Analysis Reveals Stable Inheritance and Cis-Regulation of DNA Methylation in Nasonia
Source: PLoS Biol. 2016 Jul 5;14(7):e1002500. doi: 10.1371/journal.pbio.1002500 (PMC4933354; doi:10.1371/journal.pbio.1002500)
Supplement: S1 Table — (PDF) [file pbio.1002500.s007.pdf]

OGS2_gene_id	Highly expressed species	log2FC	FDR	# of SNPs	ASE in F1VG	ASE in F1GV	Gene Annotation
Nasvi2EG018425	Ng	8.48	1.53E-53	3	0.00%	0.00%	Mitogen-activated protein kinase kinase kinase
Nasvi2EG003931	Ng	7.93	9.98E-80	2	0.00%	0.00%	harbinger transposase-derived nuclease
Nasvi2EG035843	Ng	7.53	1.20E-62	6	0.00%	0.00%	Unknown
Nasvi2EG016630	Ng	7.05	3.67E-53	5	1.88%	1.31%	hypothetical protein LOC100115888
Nasvi2EG005783	Ng	6.86	4.74E-38	2	1.96%	1.06%	venom allergen 3-like
Nasvi2EG010088	Ng	5.86	8.68E-58	6	2.45%	1.21%	Unknown
Nasvi2EG035729	Ng	5.68	9.68E-56	7	0.50%	0.96%	hypothetical protein LOC100117229
Nasvi2EG007682	Ng	5.51	3.02E-42	4	0.49%	0.00%	NADPH oxidase 5 like
Nasvi2EG000359	Ng	4.09	7.37E-18	2	8.41%	2.99%	chymotrypsin inhibitor-like
Nasvi2EG006005	Ng	4.04	3.08E-19	2	2.11%	3.40%	hypothetical protein LOC100678626
Nasvi2EG013889	Ng	2.37	9.09E-08	2	2.54%	0.00%	dynein heavy chain axonemal
Nasvi2EG017034	Nv	-1.37	0.0116	2	100.00%	100.00%	hypothetical protein LOC100679343
Nasvi2EG012837	Nv	-1.52	5.31E-06	2	96.68%	90.83%	Membrane metallo-endopeptidase 1
Nasvi2EG002495	Nv	-1.65	4.55E-07	5	98.49%	96.15%	mrna-capping enzyme-like
Nasvi2EG005683	Nv	-1.77	0.0006549	2	86.99%	86.60%	hypothetical protein LOC100678580
Nasvi2EG016161	Nv	-1.81	0.0001347	4	86.91%	85.78%	peptidoglycan recognition protein
Nasvi2EG012813	Nv	-2.03	0.0172134	4	96.93%	100.00%	Unknown
Nasvi2EG002983	Nv	-2.46	8.80E-16	4	93.73%	92.51%	hypothetical protein LOC100115681
Nasvi2EG004029	Nv	-2.46	9.58E-15	5	99.12%	100.00%	inducible metalloproteinase inhibitor protein
Nasvi2EG012455	Nv	-2.66	5.35E-17	8	100.00%	97.50%	hypothetical protein LOC100116143
Nasvi2EG012057	Nv	-2.70	1.53E-19	4	100.00%	96.68%	Cytochrome P450 4C1
Nasvi2EG025693	Nv	-2.84	1.72E-17	2	96.84%	97.16%	fatty acyl- reductase cg5065-like
Nasvi2EG006972	Nv	-2.87	3.60E-07	2	89.24%	100.00%	cytochrome p450 6k1-like
Nasvi2EG006109	Nv	-2.90	3.19E-10	5	97.79%	94.95%	Circadian clock-controlled protein
Nasvi2EG011550	Nv	-2.91	1.93E-18	2	100.00%	100.00%	hypothetical protein LOC100115668
Nasvi2EG008427	Nv	-3.01	5.10E-06	7	100.00%	100.00%	hypothetical protein LOC100679125
Nasvi2EG012869	Nv	-3.04	1.72E-09	4	100.00%	100.00%	nvu1 - fruitless readthrough protein precursor
Nasvi2EG000282	Nv	-3.06	0.0004	4	100.00%	98.76%	hypothetical protein LOC100113975
Nasvi2EG005530	Nv	-3.21	1.39E-31	6	98.78%	98.61%	neurotrimin
Nasvi2EG012405	Nv	-3.22	2.57E-12	8	96.88%	94.14%	hypothetical protein LOC100115754
Nasvi2EG016464	Nv	-3.27	4.22E-08	2	100.00%	100.00%	yellow protein
Nasvi2EG016108	Nv	-3.51	2.68E-11	2	91.01%	96.76%	carboxylesterase clade member 4
Nasvi2EG004072	Nv	-3.53	1.47E-13	4	95.29%	100.00%	double-stranded rna-specific editase adar

OGS2_gene_id	Highly expressed species	log2FC	FDR	# of SNPs	ASE in F1VG	ASE in F1GV	Gene Annotation
Nasvi2EG002674	Nv	-3.60	4.15E-11	2	100.00%	100.00%	hypothetical protein LOC100679403
Nasvi2EG008530	Nv	-3.85	2.67E-18	2	100.00%	100.00%	neutral alpha-glucosidase ab-like
Nasvi2EG013292	Nv	-4.01	1.83E-14	2	100.00%	100.00%	hypothetical protein LOC100680390
Nasvi2EG008723	Nv	-4.25	5.67E-12	6	100.00%	100.00%	hypothetical protein LOC100678275
Nasvi2EG005827	Nv	-4.60	2.93E-18	5	100.00%	100.00%	odorant binding protein 32
Nasvi2EG017109	Nv	-4.73	3.04E-45	6	100.00%	100.00%	F-box only protein 6 like
Nasvi2EG019092	Nv	-4.79	7.78E-07	3	100.00%	100.00%	venom protein m precursor
Nasvi2EG010974	Nv	-4.92	9.43E-14	9	98.10%	99.65%	hypothetical protein LOC100118583
Nasvi2EG027375	Nv	-5.25	1.52E-62	3	99.69%	100.00%	hypothetical protein LOC100678738
Nasvi2EG008724	Nv	-5.58	8.72E-28	4	100.00%	100.00%	hypothetical protein LOC100678392
Nasvi2EG017698	Nv	-5.63	1.83E-29	3	100.00%	100.00%	laccase-1
Nasvi2EG027001	Nv	-5.71	1.69E-33	2	100.00%	100.00%	hypothetical protein LOC100679921
Nasvi2EG009774	Nv	-5.93	1.20E-21	10	100.00%	100.00%	tpr domain-containing protein
Nasvi2EG002048	Nv	-6.09	1.92E-32	3	100.00%	100.00%	Unknown
Nasvi2EG007568	Nv	-6.34	1.98E-33	17	100.00%	100.00%	venom carboxylesterase-6
Nasvi2EG026476	Nv	-6.53	4.78E-53	3	100.00%	100.00%	dna pol b2 domain-containing protein
Nasvi2EG025043	Nv	-6.57	9.43E-44	3	100.00%	100.00%	hypothetical protein LOC100679011
Nasvi2EG021197	Nv	-6.77	1.39E-29	6	100.00%	100.00%	hypothetical protein LOC100680435
Nasvi2EG025958	Nv	-6.84	2.55E-50	2	100.00%	100.00%	hypothetical protein LOC100678973
Nasvi2EG031633	Nv	-7.44	7.53E-70	3	100.00%	100.00%	nuclease harbi1-like
Nasvi2EG034284	Nv	-8.11	1.10E-40	3	100.00%	100.00%	hypothetical protein LOC100678554
Nasvi2EG028731	Nv	-8.17	1.30E-48	4	100.00%	100.00%	TE:Endonuclease/Reverse transcriptase
Nasvi2EG025046	Nv	-8.19	4.41E-65	2	100.00%	100.00%	nibrin-like
Nasvi2EG013151	Nv	-8.20	6.20E-50	4	100.00%	100.00%	hypothetical protein LOC100679253
Nasvi2EG004200	Nv	-9.25	2.84E-71	6	100.00%	100.00%	serine threonine-protein phosphatase 6 regulatory subunit
Nasvi2EG025044	Nv	-9.57	6.64E-77	2	100.00%	100.00%	probable e3 ubiquitin-protein ligase trip12-like
Nasvi2EG016883	Nv	-9.77	1.97E-96	2	100.00%	100.00%	hypothetical protein LOC100115309
Nasvi2EG018142	Nv	-11.01	2.62E-119	4	100.00%	100.00%	retrovirus-related pol polyprotein from transposon tnt 1-94
